# Dynamic changes of quality of life in muscle-invasive bladder cancer survivors

**DOI:** 10.1186/s12894-022-01084-7

**Published:** 2022-08-20

**Authors:** Yuh-Shyan Tsai, Tzu-Yi Wu, Chien-Hui Ou, Hong-Lin Cheng, Tzong-Shin Tzai, Wen-Horng Yang, Jung-Der Wang

**Affiliations:** 1 Department of Urology, National Cheng Kung University Hospital, College of Medicine, National Cheng Kung University, No. 1, University Road, Tainan, 701 Taiwan; 2 Department of Occupational Therapy, Asia University, Taichung, 413 Taiwan; 3 Department of Urology, Tainan Municipal An-Nan Hospital, Tainan, 709 Taiwan; 4 Departments of Internal Medicine and Occupational and Environmental Medicine, National Cheng Kung University Hospital, Tainan, 704 Taiwan; 5 Department of Public Health, College of Medicine, National Cheng Kung University, Tainan, 701 Taiwan

**Keywords:** Quality of life, Bladder cancer, Dynamic change, Radical cystectomy

## Abstract

**Background:**

To explore the dynamic changes and effects of radical cystectomy on quality of life in muscle-invasive bladder cancer survivors.

**Methods:**

Patients with muscle-invasive bladder cancer were randomly recruited in this study. We used the World Health Organization Quality of Life-Brief questionnaire to assess consecutive patients’ quality of life. We applied kernel smoothing to illustrate the dynamic changes of the domain and item scores after treatment. Mixed-effects models were constructed to determine the effects of radical cystectomy on the scores of each item and domain of the World Health Organization Quality of Life-Brief questionnaire after controlling demographic and clinical factors.

**Results:**

We collected 397 repeated measurements of the World Health Organization Quality of Life-Brief questionnaire from 109 muscle-invasive bladder cancer patients. Forty-two of them received radical cystectomy. Patients with radical cystectomy exhibited higher levels of education, less co-morbidities (i.e., diabetes and heart diseases), but were associated with more malignancies. Construction of mixed-effects models showed patients with radical cystectomy and those with bladder sparing had similar scores in the three main domains and their items, except that of certain items of physical domain. By applying kernel smoothing method, we found that stage III–IV patients consistently showed higher scores on sleep and rest after radical cystectomy for more than 5 years. In contrast, stage II patients receiving radical cystectomy did not show a higher score on the “sleep and rest” item compared with those with bladder sparing operation.

**Conclusions:**

Radical cystectomy may result in sound sleep and rest, especially in those with stage III–IV bladder cancer.

**Supplementary Information:**

The online version contains supplementary material available at 10.1186/s12894-022-01084-7.

## Background

Bladder cancer is the ninth most frequently-diagnosed cancer worldwide, and the highest incidence rates in men are seen in countries from Southern Europe, North America, Northern Africa and Western Asia [1]. There is an unusually high incidence of bladder cancer in the black-foot-endemic area, which is located in the southwest counties of Taiwan due to chronic arsenic exposure [2]. Approximately 70–80% of bladder cancer cases have non-muscle invasive bladder cancer (NMIBC) at initial diagnosis; about 70% of them easily recur after transurethral resection, and about 15% may develop to muscle-invasive disease despite regular cystoscopic surveillance, adjuvant intravesical chemotherapy, or immunotherapy [3]. In contrast, 20–30% of bladder cancer cases initially have muscle-invasive, advanced, or metastatic tumors. Currently, the standard therapy for muscle-invasive bladder cancer (MIBC) is radical cystectomy with orthotopic neobladder substitution or other urinary diversion. As for those patients not willing or not fit for radical cystectomy, radical (or maximal) transurethral resection of bladder tumor plus with either adjuvant systemic cisplatin-based chemotherapy, radiotherapy or both, is the alternative approach. Since 2016, immune check point inhibitors has presented a challenge for the standard treatment with platinum-based chemotherapy for metastatic disease [4]. Owing to the promising therapeutic progress made in recent years, patient-reported outcomes regarding radical cystectomy or bladder-sparing tri-modality therapy continues to be a concern for both patients and urologists, and deserves more investigation and surveillance [5].

Although there are many studies reporting on the health-related quality of life (QOL) of bladder cancer patients, most of these were cross-sectional and may not be applicable for examining long-term effects [6–15]. In fact, many of them focused on the effects of different urinary diversion methods on QOL [3], and some of them failed to adjust for confounding variables [16]. Thus, there is still room for more QOL studies with longitudinal follow-up and control for various predictors. To improve our understanding and facilitate shared decision making for patients with MIBC, the aims of this study were to evaluate the long-term dynamic changes in QOL among MIBC patients who received cystectomy versus those with a preserved bladder, and to determine the associations between patients’ QOL and radical cystectomy after controlling potential confounding factors.

## Methods

### Participants

**Fig. 1:**
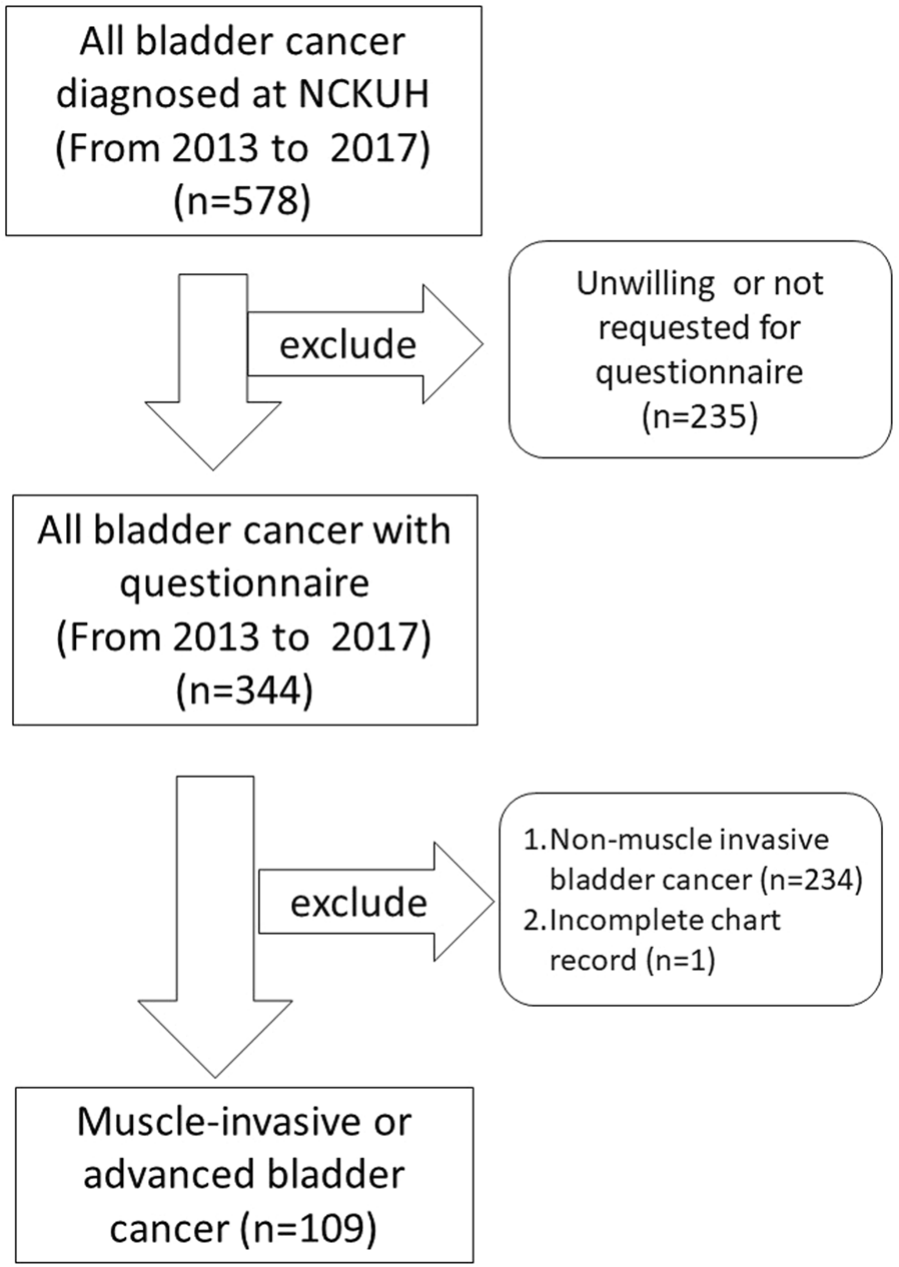
Diagram of the patients’ enrollment

This study is a single-center, retrospective study and commenced after receiving approval from the Institutional Review Board of National Cheng Kung University Hospital (A-ER-101-219). Once after obtaining the patients’ consents, bladder cancer patients who were diagnosed and/or treated at our hospital received self-reported questionnaires randomly and not consecutively on an outpatient or inpatient basis. For study convenience, we invited all the bladder cancer (both NIMBC and MIBC) patients for finishing the questionnaires. However, in the current study we just analyzed the complete questionnaires from MIBC or advanced bladder cancer, as shown in the flow chart (Fig. 1). We invited patients in all cancer stages with all kinds of treatments to participate without any exclusion. We thus included all patients with NMIBC receiving transurethral resection-bladder tumor (TUR-BT) with or without adjuvant intravesical therapy, MIBC patients undergoing radical cystectomy with various types of urinary diversions, those unwilling or unfit for cystectomy receiving bladder sparing therapy, and those with metastasis receiving salvage or palliative treatments (surgery, radiation or systemic chemotherapy. The participants were invited to repeatedly complete the WHOQOL-BREF questionnaire during the follow-up period. All the methods were performed in accordance with the Declaration of Helsinki.

### Procedures

All bladder cancer patients were invited to participate in this study by trained research assistants. The participants were instructed to self-complete the Taiwan version of the World Health Organization Quality-of-Life-Brief (WHOQOL-BREF) questionnaire via tablet computers. An experienced research assistant was available to clarify the meaning of the items in a standardized way if any questions were raised about the definition of a question or item. We also collected the patients’ demographic information (i.e., age, gender, marital status, education level, and monthly family income). Only illiterate patients took part in face-to-face interviews. The clinical data, including age at diagnosis, pathological stage, date and types of surgery, periods of systemic chemotherapy and radiotherapy, were abstracted from electronic medical records of National Cheng Kung University Hospital.

### Measurements

There were three Taiwan version of generic QOL questionnaires in Taiwan. The WHOQOL-BREF is a generic QOL questionnaire, which was developed under WHO supervision and can be utilized in general populations with or without specific diseases [17]. In contrast, the EORTC QLQ-C30 is a core generic questionnaire which was designed for disease-specific conditions (symptoms or treatment-related problems) and associated with several disease-specific modules [18]. The short form 36 health survey (SF-36) is a survey for general health and was not designed for disease-related or treatment-related problem [19]. For the potential of comparison with other diseases or conditions, WHOQOL-BREF was selected for the measurement tool. The Taiwan version of the WHOQOL-BREF contains four domains, including physical, psychological, social and environmental, and is composed of all 26 items of the WHOQOL-BREF and two local items (being respected and eating) [20]. All items are rated on a 5-point Likert scale. A higher score indicates better quality of life. The domain scores are calculated by multiplying the mean score of items in the domain by four. Thus, the score of each domain ranges from 4 to 20. The Taiwan version of the WHOQOL-BREF has been validated to have good internal consistency reliability (Cronbach’s α > 0.91), test–retest reliability (correlation coefficient > 0.75) and construct validity in patients with malignancies [20, 21].

### Statistical analysis

As patients’ pathological stages were related to the medical interventions and were likely to influence patients’ QOL, we excluded study subjects with NMIBC and focused on those with MIBC or more advanced cancers. A kernel-type smoother was used to illustrate the dynamic changes in the item and domain scores of the Taiwan version of WHOQOL-BREF using an open access software, the iSQOL software [22]. The timeline of the dynamic changes (duration-to-date for each measurement/interview) was in months after diagnosis of bladder cancer. The trends in QOL changes of patients with and without radical cystectomy were compared.

Mixed-effects models were adopted to determine the associations between the scores of each item and domain of the WHOQOL-BREF and radical cystectomy after controlling potential confounding factors, including age group (i.e., ≥ 70 years old, 60–69 years old, and < 60 years old), gender, marital status, education level, and monthly family income [i.e., more or less than 1750 US dollars (equal to 50,000 New Taiwan Dollars)], co-morbidities, clinical stage, recent chemotherapy (i.e., more or less than 12 months after the first chemotherapy), history of definite or salvage radiotherapy (i.e., yes or no) and interaction of age groups and radical cystectomy. The mixed-effects models were performed using the IBM SPSS 20 software.

## Results

### Demographics and clinical characteristics of the participants

From January 2013 to August 2017, a total of 109 muscle-invasive or advanced bladder cancer patients completed the WHOQOL-BREF with a sum of 397 repeated measurements for analysis; 42 of the patients underwent radical cystectomy (radical cystectomy + ileal neobladder, 16; radical cystectomy + ileal conduit, 22; radical cystectomy + ileal reservoir with Mitrofanoff procedure, 3; only radical cystectomy, 1). Twenty-nine patients died during the follow up. Table 1 summarizes the demographic and clinical characteristics of these participants according to the presence of an intact urinary bladder. There were no significant differences in terms of age distribution, gender, marital status and family income between patients with and without cystectomy (*p* > 0.05). As compared with cystectomized patients, cystectomized patients had higher education levels (*p* = 0.045), more synchronous/metachronous malignancy (*p* = 0.02), and less cardiac co-morbidity (*p* = 0.03). The median period from bladder cancer diagnosis to the participants’ first measurements was 14.7 months. Twenty-six of the 109 (24%) patients completed the WHOQOL-BREF only once and they had a higher mortality rate (57.7% vs. 6.0%) and higher comorbidity with heart diseases (15.4% vs. 3.6%), compared with patients who completed the questionnaire more than twice. Thirty-six (85.7%) of 42 cystectomized patients completed more than one questionnaire either before or after radical cystectomy, or both (Additional file 1: Tables S1 and S2).

**Table 1:** Demographics and clinical characteristics of bladder cancer patients stratified by pathological stage and radical cystectomy

Parameters	Bladder sparing	Radical cystectomy	*p*
Demographics			
Total no. patients (total no. measurements)	67 (211)	42 (186)	
Age (year); mean ± SD	70.0 ± 11.7	67.8 ± 8.8	0.29
Age group			0.74
≥ 70 y/o	35	18	
60–69 y/o	17	16	
< 60 y/o	15	8	
Gender (male/female)	47/20	32/10	0.49
Education (year)	7.2 ± 4.8	9.1 ± 4.7	0.04
Marital status (Married or cohabited/ others)	49/18	28/14	0.47
Monthly family income > USD. 1750 (yes/no/missing)	13/53/1	10/29/3	0.48
Clinical characteristics			
Months after the first TURBT (mean ± SD)	18.7 ± 26.8	19.1 ± 18.4	0.93
Months after the first TURBT (median [quartile deviation])	8.2 (19.6)	14.0 (18.7)	–
Stage			0.39
II	36	15	
III	9	7	
IV	22	20	
Urinary diversion			NA
Ileal neobladder	0	16	
Ileal conduit	0	22	
Ileal urinary reservoir + Mitrofanoff procedure	0	3	
None	0	1	
Comorbidity			
Diabetes mellitus	14 (20.9%)	3 (7.1%)	0.06
Heart disease	7 (10.4%)	0 (0.0%)	0.03
Other malignancy	1 (1.5%)	5 (11.9%)	0.02
Chemotherapy in the past 1 year	12 (17.9%)	4 (9.5%)	0.23
Radiotherapy	8 (11.9%)	1 (2.4%)	0.08

*SD* Standard deviation, *USD* US dollars (equal to 50,000 new Taiwan Dollars), *TUR-BT* Transurethral resection of bladder tumor, *NA* Not applicable

### Mixed-effects model for scores of domains and items

With adjusting the confounding factors, the results of mixed effect model analysis showed that female, associated with other malignancy, education year, and age more than 70 years” were independently significant worsening factors for scores of general health [estimate (standard error) of regression coefficients, − 0.30 (0.14), − 0.74 (0.26), − 0.04 (0.01), and − 0.35 (0.16), respectively]. Among the four domains, only the QOL values of physical domain and certain items were significantly influenced by these demographic and clinical characteristics (Table 2 and Additional file 2: Table S3). More advanced stage disease, associated with other malignancy, co-morbidity of heart disease, and age more than 70 years predict lower scores in the physical domain [estimate (standard error) of regression coefficients, − 0.77 (0.72), − 2.77 (0.86), − 0.99 (0.43), and, − 1.07 (0.51), respectively]. Table 2 shows the statistical results of physical domain and item scores of the WHOQOL-BREF analyzed with mixed-effect model. Briefly, advanced disease, associated with other malignancy, heart disease, more education years, and age more than 70 years exhibit negative impact on QOL values of the physical domain in muscle-invasive/advanced bladder cancer patients (Table 2 and Additional file 2: Table S3). Interestingly, male, more monthly income and radical cystectomy were favorable factors for better QOL values for sleep and rest item [estimate (standard error) of regression coefficients, 0.34 (0.14), 0.31 (0.14), and 0.36 (0.15), respectively].

**Table 2:** Regression coefficients of significant domain and item scores of the WHOQOL-BREF based on mixed-effects model

Item of WHOQOL-BREF^a, b^	Gender (Ref: female)	Monthly family income (Ref: < USD. 1750)	Stage III–IV (Ref: stage II)	Other malignancy (Ref: No)	Heart disease (Ref: No)	Educational years	Age ≥ 70 y/o	RC^c, d^ (Ref: No)
Q2_general health	*0.30 (0.14)*			− **0.74 (0.26)**		− **0.04 (0.01)**	− **0.35 (0.16)**	
Physical domain			− **1.77 (0.72)**	− **2.77 (0.86)**	− **0.99 (0.43)**		− **1.07 (0.51)**	
Q4_physical_medication				− **0.83 (0.36)**	− **0.35 (0.17)**		− **0.43 (0.22)**	
Q10_physical_energy and fatigue			− **0.62 (0.24)**	− **0.79 (0.29)**	− **0.28 (0.14)**		− **0.48 (0.17)**	
Q15_physical_mobility				− **0.98 (0.29)**	− **0.35 (0.14)**		− **0.41 (0.17)**	
Q16_physical_sleep and rest	*0.34 (0.14)*	*0.31 (0.14)*	− **0.64 (0.2)**		− **0.29 (0.12)**	− **0.04 (0.01)**		*0.36 (0.15)*
Q18_physical_work capacity	*0.33 (0.14)*			− **0.69 (0.25)**		− **0.04 (0.01)**		

Bold denotes decrease and Italic denotes increase

*USD* US dollars

QOL: Quality of life

^a^Values in parentheses are standard errors

^b^WHOQOL-BREF: World Health Organization Quality-of-Life-Brief version

^c^RC: Radical cystectomy

^d^Ref: Intact bladder with < 60 y/o

As for impact of urinary diversion types on QOL values, ileal neobladder worsened the score of the social domain as compared with did those with bladder sparing (estimate of regression coefficients, − 4.35). In contrast, either ileal conduit or partial cystectomy can improve the score of the social domain (estimate of regression coefficients, 0.62 and 2.02, respectively]. Moreover, once patients aged older than 70 years, the impact of radical cystectomy with ileal neobladder became improved in the social domain scores (Additional file 3: Table S4).

### Dynamic changes of QOL according to radical cystectomy

**Fig. 2:**
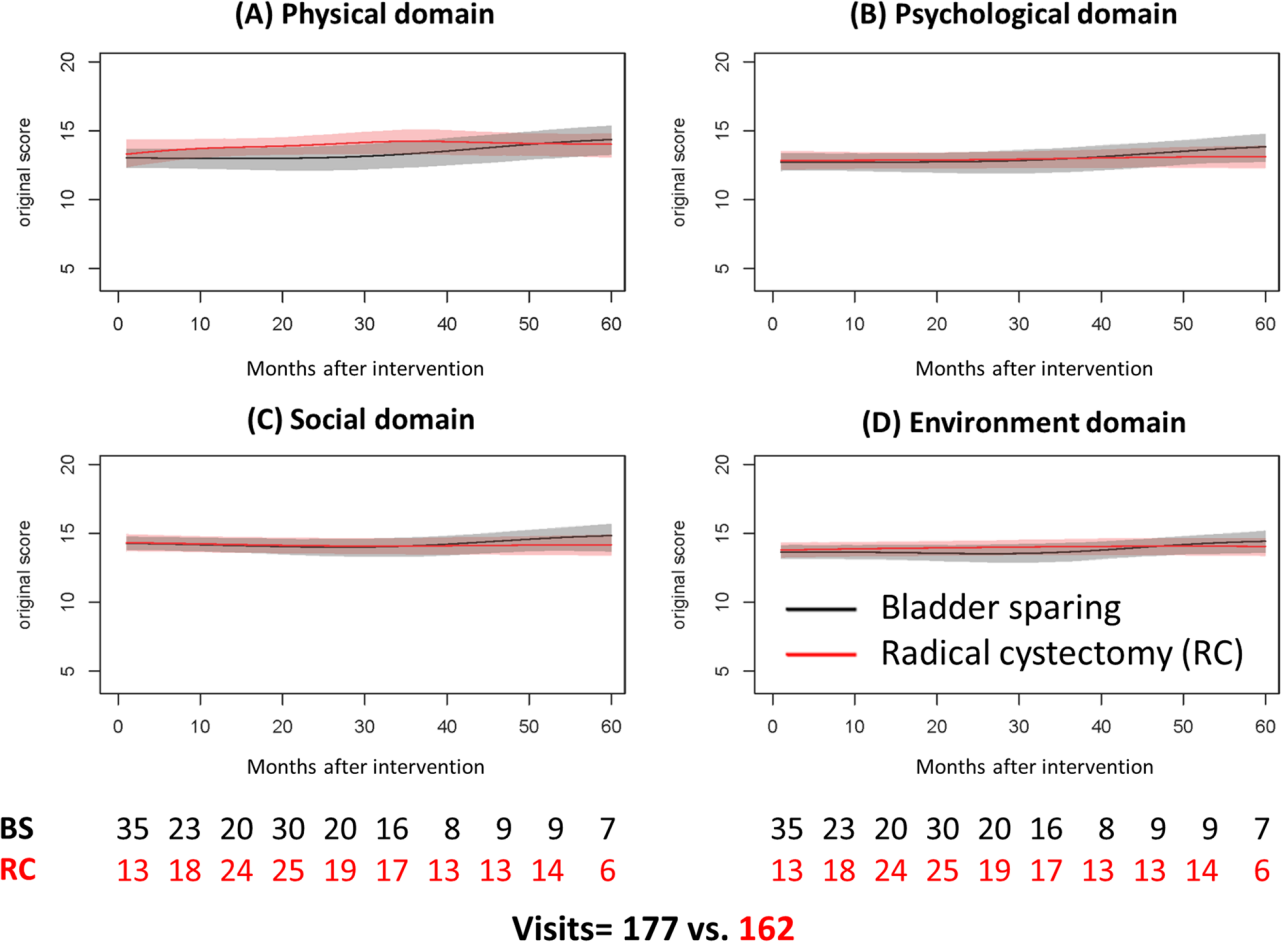
Dynamic changes of the scores of satisfactions of the 4 domains in patients with muscle-invasive bladder cancer according to treatment type. **A** Physical domain, (**B**) psychological domain, **C** social domain, **D** environmental domain. *RC* Radical cystectomy, *BS* Bladder sparing

In comparison with those receiving bladder sparing strategies, patients with radical cystectomy has better physical domain scores in the initial 4-year period, (Fig. 2A). Except this phenomenon, the curves representing the other three domain scoring were overlapped between these two groups in the initial 5 years after diagnosis (Fig. 2B–D).

**Fig. 3:**
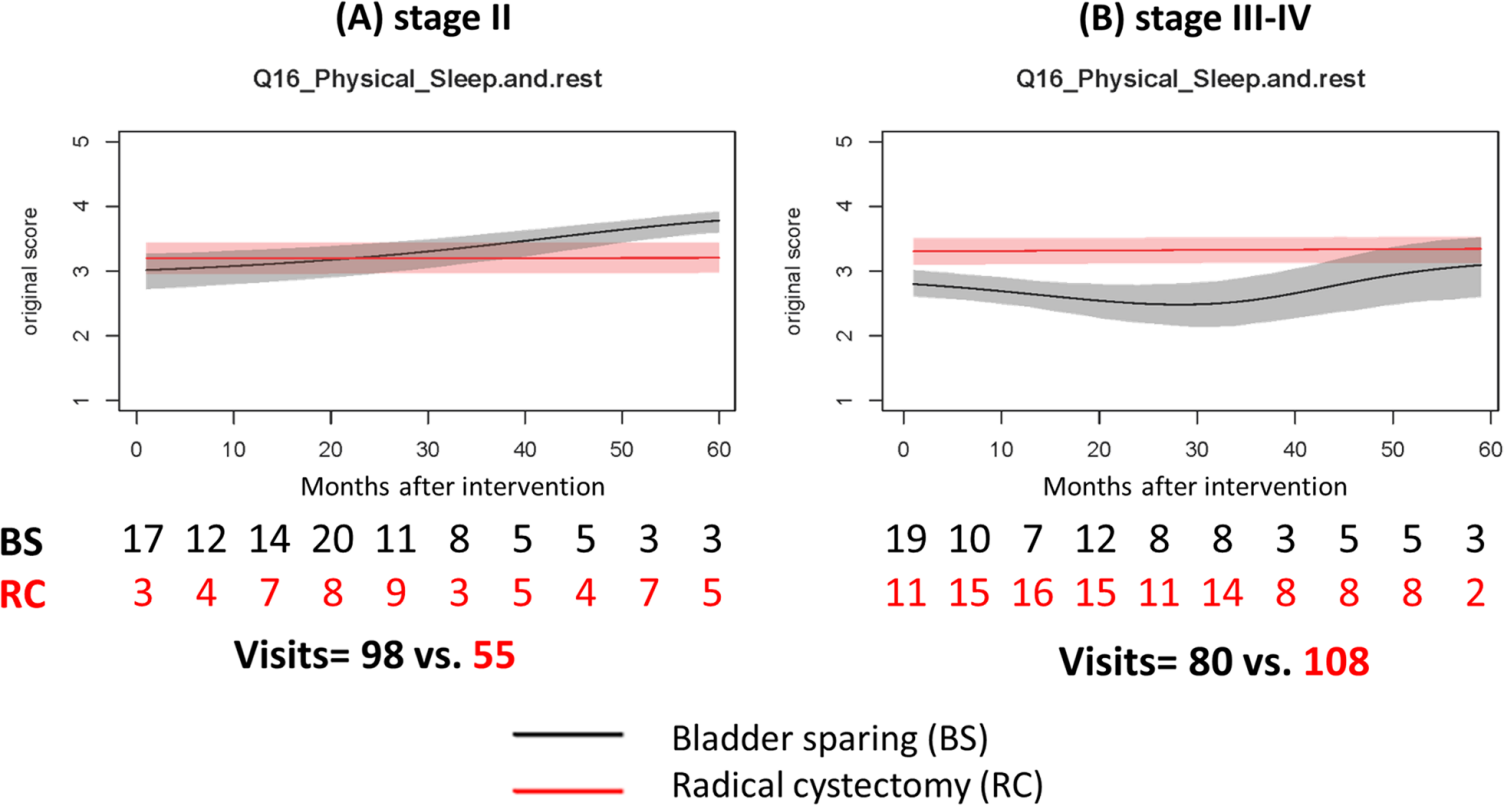
Dynamic changes of the scores of the item “sleep and rest” in patients with muscle-invasive bladder cancer. **A** Stage II patients, **B** stage III–IV patients. *RC* Radical cystectomy, *BS* Bladder sparing

Among the items of the physical domain, cystectomized patients had better scores in the item of “sleep and rest”. As for stage II patients, cystectomized patients had better scores of “sleep and rest” in the initial 2 years than the bladder sparing patients did. Later, the phenomenon reversed (Fig. 3A). As for patients in stage III or more, cystectomized patients had better scores of “sleep and rest” in the initial 5 years than the bladder sparing patients did (Fig. 3B).

## Discussion

In this study, we demonstrated that long-term patients’ concerns of generic QOL after muscle-invasive bladder cancer treatments. Except for the physical domain, muscle-invasive patients with cystectomy showed consistently similar QOL scores in the psychological, social, and environmental domains compared with those with bladder-sparing. To the best of our knowledge, little study discussed the issue regarding dynamic change. Hedgepeth RC and their colleague reported radical cystectomy has a great impact on body image (psychological domain) which improved gradually with time and regardless of urinary diversion type [23]. Despite this, determinants regarding generic QOL was not investigated. Our study also revealed that older age, meta-, or synchronous secondary malignancy, cardiovascular co-morbidity, and advanced disease can significantly worsen the physical domain score. In contrast, patients with radical cystectomy had higher scores on the item of sleep and rest for about 40 months after diagnostic TUR-BT. The reason may be because these cystectomized patients were used to using urine collection bags or diapers owing to nocturia and did not need to wake up frequently at night, whereas other people of a similar age did [24]. The above QOL results would be useful to explain the prognosis for patients with muscle-invasive bladder cancer and their family members, so that they can better prepare for the future. It is thus crucial to provide health education and consultations on personal hygiene before hospital discharge and during the patients’ clinic revisits.

This study collects longitudinal QOL data and comprehensively controls for potential confounders from different demographic and clinical factors through mixed-effects models. We found that heart disease and diabetes mellitus (though not statistically significantly) seemed to be more common in the bladder sparing group. This might be because patients with heart disease and diabetes mellitus might be less tolerant of undergoing operations and choose to keep the bladder. Moreover, cystectomized patients had more years of education than the bladder sparing patients did, which may reflect the different sense of illness, and decision making. We believe that our data reflects reality and displays an acceptable representation of the population of patients with muscle-invasive bladder cancer. Our results demonstrating the negative effects of old age and advanced disease were also consistent with previous cross-sectional studies [5, 25]. In other words, these findings corroborate the validity of our models. The findings of this study are not only useful for clinicians to explain the possible prognoses of their patients, but also serve as an initial platform for shared decision making and help optimize the value of different interventions. Despite this, it should be utilized carefully because the current study is a small number of patients, single center, retrospective analysis.

Analysis using the SEER database shows that bladder cancer can occur in 0.5% of patients with other malignancies [26] and 16.4% of bladder cancer patients develop second primary malignancies [27]. In the current study, we also found that patients with radical cystectomy were more metachronous or synchronous with other malignancy than those with bladder sparing were. These malignancies include incidental prostate cancer detection in the surgical specimen in males and the presence of upper tract urothelial carcinoma. Patients associated with other malignancy have significantly lower scores of the physical domain and its items. Although it is not easy to differentiate which type of malignancy influences QOL more, our data displays a long-term effect on QOL with controlling confounders. We only found one cross-sectional study that showed cancer survivors with more than one malignancy experienced modest, persistent declines in global QOL after controlling demographic, medical, and psychosocial characteristics [28]. Therefore, healthcare professionals must pay more attention to these issues and make multi-specialty treatment team earlier for treating such patients.

Our data showed that radical cystectomy can provide a long-lasting effect on QOL stability in the item of “sleep and rest” regardless of disease stage. In contrast, advanced bladder cancer patients with bladder preserving may suffer from pain or voiding difficulty, which results in a significant, lasting QOL decrement in “sleep and rest”. Although evidence shows trimodality bladder sparing therapy for muscle invasive bladder cancer is not inferior to standard radical cystectomy [29]; loco-regional recurrence with or without pain may frequently occur in the bladder, even after chemoradiotherapy [30]. Furthermore, some patients may suffer from late pelvic toxicity after bladder-sparing therapy, such as radiotherapy [31]. Thus, our data can provide evidence for supporting the option of radical cystectomy in MIBC patients with intolerable voiding problems or pain. In contrast, Miyanaga and their colleagues using Japanese version of EORTC QLQ-C30 questionnaire reported that sleep problems were more frequent in the radical cystectomy groups than those with bladder sparing [32]. Singer et al. using the EORTC QLQ-C30 questionnaire in a cohort of 823 bladder cancer patients demonstrated that sleep problem bothered aged ≥ 70 years male patients with ileal conduit more than those without conduit. There was no difference by sex, conduit and time length after surgery in aged less than 70 years [33]. It is difficult to make any conclusion based on these studies because there were a lot of heterogeneity among them.

There are five limitations in this study. First, all the participants were retrospectively enrolled from single medical center. The studied cohort is a heterogeneous patient population, including patients who needed palliative cystectomy and exhibited poor quality of life and very limited life expectancy (usually) after surgery. The bladder sparing group might also include some patients with poor generalized conditions, containing with some comorbidities. Such a heterogeneity could influence the generalizability. For reducing the bias, we used mixed effect model to analyzing the determinants of generic quality of life. Second, some patients were de novo muscle-invasive bladder cancer, and some patients progressed to muscle-invasive or even metastasis after experiencing a more-or-less time of non-muscle-invasive bladder cancer, which reflected from longer intervals from the diagnosis to the completion of questionnaires. Thus, such an unequal distributions of questionnaire completion existed in the current study, resulting in an analysis bias. Our result should be carefully interpreted and applied in practice. Third, there was a selection bias existing in the current study. This study is not consecutive for all diagnosed and treated patient at our hospital. Except for one patient with incomplete chart record, those unwilling or not requested for complete the questionnaire were excluded, which may have resulted in a selection bias. Fourth, we simply categorized the patient's cystectomy group into different subgroups to explore the impact of urinary diversion type on QOL. Owing to limited sample size, we just found that the potential impact on social domain, as shown in Additional file 3: Table S4. To obtain a more conclusive viewpoint, more studied subjects are needed. Fifth, our study provided an information of determinants and dynamic changes of generic QOL in muscle-invasive/advanced bladder cancer. The majority of the literature investigate the change of specific functions (i.e., bowel function or sexual function). Therefore, it is difficult to perform a direct and specific comparison of analyzed results with other studies in the literature.

## Conclusions

Our study found that older age, association with other malignancies, cardiovascular co-morbidity, and advanced disease can significantly influence the physical domain score in patients with muscle-invasive bladder cancer. Patients with radical cystectomy had higher scores on the item of “sleep and rest” for about 40 months after diagnosis than those with bladder sparing. The number of patients in the current study is small so that our conclusion should be carefully applied in the daily practice. More studies among different countries/regions enrolling more patients to clarify the limitations are warranted in the future.

## Supplementary Information

**Additional file 1. Table S1.** Comparison of demographics and clinical characteristics of bladder cancer patients who were interviewed once and more than once. **Table S2.** Number of visits in each time point.

**Additional file 2. Table S3.** Regression coefficients of each domain and item scores of the WHOQOL-BREF based on mixed-effects model in stage II and III MIBC patients.

**Additional file 3. Table S4.** Effect of urinary diversion type on values of social domain score.

## Data Availability

The data that support the findings of this study are available from rom electronic medical records and clinical cancer center of National Cheng Kung University Hospital, but restrictions apply to the availability of these data, which were used under license for the current study, and so are not publicly available. Data are however available from the authors upon reasonable request and with permission of the Institutional Review Board of National Cheng Kung University Hospital.

## Abbreviations

NMIBC: Non-muscle invasive bladder cancer
MIBC: Muscle-invasive bladder cancer
QOL: Quality of life
WHOQOL-BREF: World Health Organization Quality-of-Life-Brief
TUR-BT: Transurethral resection of bladder tumor
