# Supplementary material for: Dynamic changes of quality of life in muscle-invasive bladder cancer survivors
Source: BMC Urol. 2022 Aug 20;22:126. doi: 10.1186/s12894-022-01084-7 (PMC9392945; doi:10.1186/s12894-022-01084-7)
Supplement: Supplementary file 1 — Additional file 1. Table S1. Comparison of demographics and clinical characteristics of bladder cancer patients who were interviewed once and more than once. Table S2. Number of visits in each time point. [file 12894_2022_1084_MOESM1_ESM.doc]

**Supplementary Table 1**. Comparison of demographics and clinical characteristics of bladder cancer patients who were interviewed once and more than once

|  | one interview | ≥ 2 interviews | *p value* |
| --- | --- | --- | --- |
| Total no. patients (measurements) | 26 (26) | 83 (371) |  |
| Age (year); Mean ± SD | 72.4±11.1 | 68.0±10.4 | 0.06 |
| Age group |  |  | 0.05 |
| ≥70 y/o | 18 (69.3%) | 35 (42.2%) |  |
| <70 y/o | 8 (30.0%) | 48 (57.87%) |  |
| Vital status (dead) | 15 (57.7%) | 5 (6.0%) | <0.01 |
| Radical cystectomy, N | 6 (14.3%) | 36 (85.7%) |  |
| Ileal neobladder | 1 | 15 | 0.08 |
| Ileal conduit | 3 | 19 |  |
| Others | 2 | 2 |  |
| Stage |  |  | 0.36 |
| 2-3 | 14 (53.9%) | 53 (63.9%) |  |
| 4 | 12 (46.2%) | 30 (36.1%) |  |
| Comorbidity |  |  |  |
| Diabetes mellitus | 5 (19.2%) | 12 (14.5%) | 0.56 |
| Heart disease | 4 (15.4%) | 3 (3.6%) | 0.03 |
| Other malignancy | 0 (0.0%) | 6 (7.2%) | 0.16 |

SD, standard deviation

**Supplementary Table 2. Number of visits in each time point**

| Months after first diagnostic TUR-BT | Bladder sparing | Radical cystectomy |
| --- | --- | --- |
| 0.0-12.0 | 58 | 31 |
| 12.1-24.0 | 51 | 48 |
| 24.1-36.0 | 35 | 37 |
| 36.1-48.0 | 18 | 26 |
| 48.1-60.0 | 15 | 20 |
| > 60.0 | 34 | 24 |
| Total | 211 | 186 |

TUR-BT, transurethral resection of bladder tumor
