# Supplementary material for: Dynamic changes of quality of life in muscle-invasive bladder cancer survivors
Source: BMC Urol. 2022 Aug 20;22:126. doi: 10.1186/s12894-022-01084-7 (PMC9392945; doi:10.1186/s12894-022-01084-7)
Supplement: Supplementary file 2 — Additional file 2. Table S3. Regression coefficients of each domain and item scores of the WHOQOL-BREF based on mixed-effects model in stage II and III MIBC patients. [file 12894_2022_1084_MOESM2_ESM.doc]

Supplementary Table 3. Regression coefficients of each domain and item scores of the WHOQOL-BREF based on mixed-effects model in stage II and III MIBC patients

| Item of WHOQOL-BREF | Age ≥70 years^a^ | Male^b^ | Monthly family income^c^ | Education year^d^ | Heart Disease^e^ | Diabetes^f^ | Other malignancies^g^ | RC^h^ | Chemotherapy^i^ | |  |
| --- | --- | --- | --- | --- | --- | --- | --- | --- | --- | --- | --- |
| Q1_Overall QoL | -0.38(0.16) |  |  |  |  |  | -0.71(0.32) |  |  | |  |
| Q2_General health | -0.56(0.17) | 0.34(0.16) |  |  |  |  |  |  |  | |  |
| **Physical Domain** | -1.73(0.5) |  |  |  |  |  | -2.7(0.96) |  |  | |  |
| Q3_Physical_Pain and discomfort | -0.39(0.18) | 0.38(0.18) |  |  |  |  |  |  |  | |  |
| Q4_Physical_Medication |  |  |  |  |  | -0.87(0.27) |  |  |  | |  |
| Q10_Physical_Energy and fatigue | -0.54(0.17) |  |  |  |  |  | -1.3(0.35) |  |  | |  |
| Q15_Physical_Mobility | -0.66(0.18) |  |  |  |  |  |  |  | 0.46(0.23) | |  |
| Q16_Physical_Sleep and rest | -0.39(0.18) |  |  | -0.06(0.02) |  |  | -0.9(0.35) |  |  | |  |
| Q17_Physical_Activities of daily living | -0.35(0.14) |  |  | -0.03(0.01) |  |  |  |  |  | |  |
| Q18_Physical_Work capacity |  | 0.39(0.16) |  | -0.03(0.02) |  |  |  |  |  | |  |
| **Psychological Domain** | -1.35(0.53) |  |  |  |  |  |  |  |  | |  |
| Q5_Psychological_Positive feelings | -0.49(0.18) |  |  |  |  |  |  |  |  | |  |
| Q6_Psychological_Spirituality / religion / beliefs | -0.53(0.18) |  |  |  |  |  |  | -0.42(0.18) | |  | |
| Q7_Psychological_Concentration |  |  |  |  |  |  |  |  |  | |  |
| Q11_Psychological_Body image |  |  |  |  |  |  |  |  |  | |  |
| Q19_Psychological_Self-esteem |  |  |  |  |  |  |  |  |  | |  |
| Q26_Psychological_Negative feelings |  |  |  |  |  |  | -0.97(0.36) |  |  | |  |
| **Social Domain** |  |  |  |  |  |  | -2.43(0.98) |  |  | |  |
| Q20_Social_Personal relationship | -0.25(0.13) |  |  | -0.02(0.01) |  |  |  |  |  | |  |
| Q21_Social_Sexual activity |  |  |  |  |  |  | -1(0.41) |  |  | |  |
| Q22_Social_Social support | -0.27(0.13) |  |  |  |  |  |  |  | 0.37(0.19) | |  |
| Q27_Social_Being respected |  |  |  |  |  |  |  |  |  | |  |
| **Environment Domain** |  |  | 1.23(0.56) |  |  |  |  |  |  | |  |
| Q8_Environment_Safety and security |  |  |  |  |  |  |  |  |  | |  |
| Q9_Environment_Physical Environmental |  |  |  | -0.03(0.01) |  |  |  |  |  | |  |
| Q12_Environment_Financial resources |  |  | 1.07(0.25) |  | 1.03(0.48) |  |  |  |  | |  |
| Q13_Environment_Information acquiring |  |  |  |  |  |  |  |  |  | |  |
| Q14_Environment_Leisure activities |  |  |  |  |  |  |  |  |  | |  |
| Q23_Environment_Home Environmental |  |  |  | -0.03(0.01) |  |  |  |  |  | |  |
| Q24_Environment_Health services |  |  |  |  |  |  |  |  |  | |  |
| Q25_Environment_Transportation |  |  |  |  |  |  |  |  |  | |  |
| Q28_Environment_Eating |  |  |  |  |  |  |  |  |  | |  |

Values in parentheses are standard errors.

^a^Age (≥70 years versus <70 years); ^b^Male (Male versus Female); ^c^Monthly family income (> USD. 1750 versus <1750); ^d^education years (continuous variable); ^e^Heart disease (Yes versus No); ^f^Diabetes (Yes versus No); ^g^other malignancies (Yes versus No); ^h^RC, Radical cystectomy (Yes versus No); ^i^recent chemotherapy (Yes versus No)

QOL, Quality of Life; WHOQOL-BREF, World Health Organization Quality-of-Life-Brief version
