# Supplementary material for: Dynamic changes of quality of life in muscle-invasive bladder cancer survivors
Source: BMC Urol. 2022 Aug 20;22:126. doi: 10.1186/s12894-022-01084-7 (PMC9392945; doi:10.1186/s12894-022-01084-7)
Supplement: Supplementary file 3 — Additional file 3. Table S4. Effect of urinary diversion type on values of social domain score. [file 12894_2022_1084_MOESM3_ESM.docx]

| Social domain | Regression coefficients (β) |
| --- | --- |
| Ileal neobladder^a^ | -4.35* |
| Ileal conduit^a^ | 0.63 |
| Partial cystectomy^a^ | 2.02 |
| Ileal neobladder (and ≥ 70 years)^b^ | 4.73* |

Supplementary Table 4. Effect of urinary diversion type on values of social domain score

^a^Bladder sparing as reference; ^b^Bladder sparing (and < 70 years) as reference;*denotes statistically significant
